# Molecular mechanisms for simvastatin-mediated enhancement of synaptic plasticity

**DOI:** 10.1186/1750-1326-7-S1-O4

**Published:** 2012-02-07

**Authors:** Robert A Mans, Lori L McMahon, Ling Li

**Affiliations:** 1 Neurobiology, University of Alabama at Birmingham, Birmingham, Alabama, USA; 2 Medicine, University of Alabama at Birmingham, Birmingham, Alabama, USA; 3 Physiology and Biophysics, University of Alabama at Birmingham, Birmingham, Alabama, USA; 4 Department of Experimental and Clinical Pharmacology, University of Minnesota, Minneapolis, Minnesota, USA

## 

Simvastatin (SV), a competitive inhibitor of 3-hydroxy-3-methylglutaryl CoA reductase and a widely prescribed treatment for hypercholesterolemia, exerts numerous positive pleiotropic effects that are thought to occur independent of its cholesterol-lowering properties. In previously published work, we have shown that chronic SV treatment rescues cognitive function in a transgenic mouse model of Alzheimer’s Disease, and enhances learning and memory in non-transgenic mice without affecting total brain cholesterol and amyloid-beta levels. More recently, we demonstrated the ability of SV to enhance long-term potentiation (LTP) in the CA1 region of the hippocampus in slices from wild type C57BL/6 mice via a mechanism dependent upon phosphatidylinositol 3-kinase (PI3-K)/Akt activation during LTP induction. The present study was conducted to better understand the molecular mechanisms underlying SV-induced enhancement of LTP. Specifically, it was found that inhibiting production of isoprenoid intermediates in the biosynthetic pathway for cholesterol triggers the downstream events leading to enhanced LTP. Interestingly, two major isoprenoid intermediates exhibit differential effects. Replenishment of farnesyl pyrophosphate, but not geranylgeranyl pyrophosphate, abolished the LTP-enhancing ability of SV. Parallel to this finding, inhibiting farnesylation, but not geranylgeranylation replicated the enhancement of LTP caused by SV. Finally, inhibiting farnesylation promotes the activation of Akt during the induction phase. Together, these results suggest that SV enhances LTP in CA1 by modulating isoprenylation-dependent molecular pathways downstream of farnesyl transferase. These findings will aid in the identification of novel therapeutic targets that modulate synaptic and cognitive function.

*Grant support: Supported by grants of NIH AG031846 and an Anonymous Philanthropic Foundation to L Li*.

### Acknowledgements

This study was supported by Program for Excellent talent in University of Chongqing (2010).

